# Combined effect of sonophoresis and a microemulsion on the dermal delivery of celecoxib

**DOI:** 10.1080/10717544.2020.1797244

**Published:** 2020-07-24

**Authors:** Thirapit Subongkot

**Affiliations:** Faculty of Pharmaceutical Sciences, Department of Pharmaceutical Technology, Burapha University, Chonburi, Thailand

**Keywords:** Microemulsion, celecoxib, PEG-6 caprylic/capric glycerides, sonophoresis, dermal drug delivery

## Abstract

The aim of this study was to evaluate the intensity of sonophoresis at which the skin penetration of celecoxib was enhanced and to study the combined effects of sonophoresis and microemulsion application on the dermal delivery of celecoxib. The sonophoresis intensity that provided the highest skin penetration enhancement of celecoxib was 30 Watts/cm^2^. However, the combination of sonophoresis and the microemulsion resulted in a decrease in celecoxib skin penetration. The results of a confocal laser scanning microscopy study using the colocalization analysis of multifluorescently labeled particles revealed that the reduction in skin penetration of celecoxib from the combination of sonophoresis and a microemulsion resulted from a decrease in transfollicular penetration, which is the major skin absorption pathway of the microemulsion.

## 1. Introduction

Celecoxib, a nonsteroidal anti-inflammatory drug (NSAID) that specifically inhibits cyclooxygenase-2 enzyme (COX-2), has been approved for the treatment of acute pain, rheumatoid arthritis, and osteoarthritis, as well as primary dysmenorrhea. Among NSAIDs, celecoxib has been widely used due to its lower risk of upper gastrointestinal tract bleeding. However, the use of celecoxib could increase the risk of cardiovascular events and renal failure (Ahmad et al., 2002; Caldwell et al., 2006). The serious side effects of celecoxib, such as myocardial infarction and acute kidney injury, occur through oral absorption to the systemic circulation. Therefore, the development of celecoxib for topical delivery is necessary to avoid these adverse events.

Topical administration, the direct delivery of a drug to the target site, can be used alternatively to oral administration. Topical administration offers advantages over oral administration, such as avoiding hepatic first-pass metabolism and reducing the amount of drug in the systemic circulation, which leads to a lower incidence of adverse events. Topical administration of celecoxib is the delivery of celecoxib through the skin to the inflammation area located in a muscle, bone, or joint. The efficacy of the treatment depends on the amount of drug that successfully penetrated to the inflammation area. However, the percutaneous absorption of drugs is limited by the stratum corneum, the outermost layer of the skin. Generally, the physicochemical properties of molecules that can simply penetrate the skin should have a log partition coefficient (Log P) between 1 and 3 with a molecular weight of no more than 500 Da (Naik et al., 2000). Celecoxib, a highly lipophilic drug, has a log P of 3.53 with a molecular weight of 381.4 Da (Drugbank, 2019). Thus, celecoxib exhibits poor percutaneous absorption, and the development of a percutaneous absorption enhancement technique is needed to improve the amount of celecoxib at target sites.

Sonophoresis is a physical technique that applies low-frequency ultrasound (18-100 kHz) to increase skin penetration (Herwadkar et al., 2012). Many reports have shown that sonophoresis can increase the skin penetration of many drugs, such as ketoprofen (Herwadkar et al., 2012), heparin (Mitragotri & Kost, 2001), niacinamide (Park et al., 2019), and minoxidil (Park et al., 2019). Although sonophoresis could enhance skin penetration, the corresponding mechanisms have still not been evaluated. It has been proposed that the enhancement mainly results from cavitation effects (Azagury et al., 2014; Park et al., 2014). It has also been suggested that the stratum corneum barrier is weakened by small bubbles generated from sonophoresis.

Microemulsions are transparent colloidal systems composed of the oil phase, a surfactant mixture, and water phase. Microemulsions offer many advantages, such as thermodynamically stable systems, ease of preparation, and a high solubilization capacity for both hydrophilic and lipophilic drugs (Spernath & Aserin, 2006; He et al., 2010). There is a report showing that a microemulsion could enhance skin penetration of celecoxib (Subongkot & Sirirak, 2020). It is suggested that the mechanism of skin penetration enhancement of microemulsion resulted from increased transfollicular penetration (Subongkot & Sirirak, 2020) according to the different mechanisms of percutaneous penetration enhancement of combined sonophoresis and a microemulsion. Therefore, this study initiated the application of sonophoresis and microemulsions to improve the skin penetration of celecoxib, which has not been reported elsewhere.

This study aimed to evaluate the optimum sonophoresis intensity for enhanced skin absorption of celecoxib and to investigate the combined effects of sonophoresis and a microemulsion on the skin penetration of celecoxib.

## 2. Materials and methods

### 2.1. Materials

Celecoxib was purchased from Power Tech Chemical Industry, Bangkok, Thailand. Isopropyl myristate (IPM) was purchased from Namsiang Co., Ltd. Bangkok, Thailand. PEG-6 caprylic/capric glycerides (Cetiol 767) were purchased from Basf Personal Care and Nutrition GmbH, Monheim, Germany. PEG-7 glyceryl cocoate (Cetiol HE) was purchased from Basf Personal Care and Nutrition GmbH, Monheim, Germany. Solvent red 49 or rhodamine B base was purchased from Sigma Aldrich, St. Louis, MO, USA. 1,2-Dihexadecanoyl-sn-glycero-3-phosphoethanolamine triethylammonium salt (NBD-PE) was purchased from Thermo Fisher Scientific, Waltham, MA, USA. All other reagents were of analytical grade and were commercially available.

### 2.2. Effect of sonophoresis intensity on the skin penetration of celecoxib

#### 2.2.1. Preparation of a celecoxib solution

Celecoxib was accurately weighed in a glass vial and dissolved and adjusted with PEG 400 to provide a concentration of 2% w/w.

#### 2.2.2. Skin preparation

Full-thickness porcine skin was obtained from the abdominal part of a neonatal pig that died naturally and was provided by a local farm. After cutting the skin from the body, the muscle layers were carefully removed from the skin by a surgical blade in which the skin thickness was not more than 1 mm. The prepared skin was mounted between the donor and receiver chambers of a Franz diffusion cell with the stratum corneum facing the donor chamber. The donor chamber was filled with 1.5 ml of distilled water as the coupling medium.

#### 2.2.3. Sonophoresis intensity calculation

The skin was treated with various sonophoresis intensities (6, 8, 10, 15, 30, 60, 90 and 120 Watts/cm^2^) prior to the *in vitro* skin penetration study. The sonophoresis intensity was calculated using the following equation:

(1) W=J/(A×T)

*W* = sonophoresis intensity (Watts/cm^2^)
*J* = energy (Joules)
*A* = penetration area of the Franz diffusion cell (cm^2^)
*T* = sonophoresis application time (s)

#### 2.2.4. Sonophoresis application and *in vitro* skin penetration study

This sonophoresis study was performed using low-frequency ultrasound (20 kHz) generated by a probe sonicator (Vibra-Cell^TM^ VCX 750, Sonics & Materials INC., Newtown, CT) that was compared to passive penetration as a control. The probe sonicator tip (6 mm in diameter) was immersed in the coupling medium inside the donor chamber of the Franz diffusion cell mounted with porcine skin as described in Section 2.2.2 with a distance between the tip and the skin of 3 mm. The skin was treated with different sonophoresis intensities according to Section 2.2.3 for 2 min. Then, the coupling medium was discarded, and the skin was wiped with tissue paper to completely remove any remaining coupling medium. Afterward, the Franz diffusion cell was connected with water circulating bath to maintain the temperature at 37 ± 0.2 °C, and the receiver compartment was filled with approximately 6.5 ml of 50% v/v ethanol in PBS and stirred gently with a magnetic stir bar. The donor chamber was filled with a celecoxib solution prepared as described in Section 2.2.1 and covered with Parafilm^®^. After treatment for 6 h, the skin was removed from the Franz diffusion cell, and the celecoxib amount was subsequently determined. The receiver medium was withdrawn, filtered with a nylon syringe filter, and analyzed for celecoxib concentration by HPLC.

#### 2.2.5. Determination of celecoxib concentration in the skin

The amount of celecoxib in the treated skin was determined using the tape stripping technique as described by Subongkot and Sirirak (Subongkot & Sirirak, 2020). The skin was fixed with pins on a tray containing hard paraffin. The stratum corneum side of the skin was wiped with tissue paper to remove any remaining celecoxib solution. Then, the stratum corneum layers were removed by stripping with 24 mm wide pressure-sensitive adhesive tape (Scotch^®^ Transparent Tape 500, 3 M Co., Ltd., Bangkok, Thailand) for 35 times, which covered the whole penetration area. All stripped adhesive tape pieces were transferred into a tightly closed screw-top glass vial containing 5 ml of methanol and sonicated with a sonicator bath (WUC-D22H, DAIHAN Scientific, Gang-won-do, Korea) for 15 min. Then, 1 ml of methanol was pipetted into the microcentrifuge tube and centrifuged at 11,180 × *g* at 25 °C for 15 min (Thermo Scientific™Sorvall^TM^Legend^TM^ XTR Centrifuge, Thermo Scientific^TM^, Waltham, MA). The obtained supernatant was quantitatively analyzed for celecoxib by high-performance liquid chromatography (HPLC). The celecoxib amount in the skin was calculated from Equation (2) as follows:

(2) $$\text{Drug}\;\text{amount}\;\text{in}\;\text{the}\;\text{stratum}\;\text{corneum}\;\left(\mu g/{\text{cm}}^{2}\right)\;=Cs/A$$

*C*s = amount of celecoxib in the stratum corneum (µg)
*A* = skin absorption area (cm^2^)

The skin from which the stratum corneum was removed, cut into small pieces and immersed in 3 ml of methanol in a tightly closed screw cap glass vial before being sonicated in the sonicator bath for 15 min. The obtained supernatant solution was, subsequently, analyzed by HPLC. The amount of celecoxib in the tissue (viable epidermis and dermis) was calculated from Equation (3) as follows:

(3) $$\text{Drug}\;\text{amount}\;\text{in}\;\text{the}\;\text{viable}\;\text{epidermis}\;\text{and}\;\text{dermis}\;\left(\mu g/{\text{cm}}^{2}\right)\;=Cv/A$$

*C*v = amount of celecoxib in the viable epidermis and dermis (µg)
*A* = skin absorption area (cm^2^)

### 2.3. Combined effects of a microemulsion and sonophoresis on the skin penetration of celecoxib

#### 2.3.1. Preparation of celecoxib-loaded microemulsion

According to the study by Subongkot and Sirirak (Subongkot & Sirirak, 2020), the authors found that a 2% w/w celecoxib-loaded microemulsion composed of isopropyl myristate (IPM) as the oil phase, PEG-6 caprylic/capric glycerides:PEG-7 glyceryl cocoate (1:1 w/w) as the surfactant:cosurfactant and water as the aqueous phase at an oil:surfactant mixture:water ratio of 0.1:0.6:0.3 could significantly improve the skin penetration of celecoxib when compared to celecoxib in PEG 400 as a control. This study, therefore, selected the microemulsion formulation as described above to investigate its combined effects with sonophoresis. To prepare the 2% w/w celecoxib loaded microemulsion, IPM, a surfactant mixture, and water were weighed into glass vials according to the proportions described above and stirred for 10 min with a magnetic stir bar. Then, the obtained blank microemulsion was transferred to a glass vial containing celecoxib to provide a drug concentration of 2% w/w. Afterward, the suspension was placed in the sonicator bath for 10 min until celecoxib was completely dissolved.

#### 2.3.2. Combined effects of the microemulsion and sonophoresis

To study the combined effect of sonophoresis and the microemulsion, porcine skin was pretreated with sonophoresis before treatment with the microemulsion. Porcine skin was mounted in the Franz diffusion cell and treated with sonophoresis under the same conditions as described in Section 2.2.4 using the sonophoresis intensity which provided the highest skin penetration of celecoxib. After the skin was treated with sonophoresis and the coupling medium was discarded, the donor compartment was filled with 2 ml of the 2% w/w celecoxib microemulsion as prepared in Section 2.3.1. The skin penetration study was performed using the same conditions as described in Section 2.2.4. The analysis of the amount of celecoxib was performed using the same methods as described in Section 2.2.5.

### 2.4. Transfollicular penetration study

Among reported skin penetration pathways, transfollicular penetration is the major pathway of microemulsions (Subongkot & Sirirak, 2020). To investigate the mechanism of increased or decreased celecoxib skin penetration resulting from the combination of sonophoresis and the microemulsion, confocal laser scanning microscopy (CLSM) using a colocalization technique was used to visualize the skin penetration of fluorescently labeled microemulsion particles. Rhodamine B base, a lipophilic molecule that has a log P value (log P = 1.95) close to that of celecoxib and exhibits red fluorescence, was utilized as the entrapped drug. To track the microemulsion particle, NBD-PE, a phospholipid surfactant conjugated with a green fluorescent molecule, was used to probe the microemulsion particles with fluorescent color against the entrapped drugs.

#### 2.4.1. Preparation of a rhodamine B base-loaded, NBD-PE-labeled microemulsion

Rhodamine B base and NBD-PE were accurately weighed with an analytical balance to 10 mg and 5 mg, respectively, in a 2 ml volumetric test tube. The blank microemulsion was placed into a volumetric test tube before being placed in a sonicator bath until rhodamine B base including NBD-PE were dissolved. Then, an additional blank microemulsion was filled to adjust the final volume to 2 ml.

#### 2.4.2. Skin penetration study

The skin penetration study was performed with the Franz diffusion cell except for the presence of a receiving medium. The skin was pretreated with the sonophoresis intensity that provided the highest celecoxib skin penetration. Then, the coupling medium was discarded, and 300 µl of the rhodamine B base-entrapped, NBD-PE-labeled microemulsion was placed in the donor chamber. After treatment for 2 h, the microemulsion was withdrawn and the skin was washed with PBS to remove any excess dyes. The treated skin was immediately observed with CLSM.

#### 2.4.3. Confocal laser scanning microscopy (CLSM) study

The treated skin obtained in Section 2.4.2 was put down on a 22 × 50 mm^2^ coverslip (MENZEL-GLÄSER^®^, Braunschweig, Germany) by turning the stratum corneum to a 10× objective lens of an inverted confocal laser scanning microscope (Zeiss LSM 800, Carl Zeiss, Jena, Germany) equipped with solid-state diode lasers. The red fluorescence of rhodamine B base was determined at excitation and emission wavelengths of 577 and 603 nm, respectively. The green fluorescence of NBD-PE was determined at excitation and emission wavelengths of 493 and 517 nm, respectively.

To compare the fluorescence intensity of the x-z plane images, the skin was visualized, and images were taken by a 20× objective lens. The fluorescence intensity was determined from the middle horizontal line of each image using Zeiss Zen microscope software (Zen lite, blue edition). The average fluorescence intensity was plotted against the skin penetration depth.

### 2.5. High-performance liquid chromatography (HPLC) analysis

Celecoxib was quantitatively analyzed using HPLC (Agilent 1260 infinity II LC system, Agilent Technology, Santa Clara, CA) with a 4.6 mm × 250 mm C18 reversed-phase column containing a particle size of 5 µm (VertiSep UPS C18, Vertical, Nonthaburi, Thailand) according to the validated method of Subongkot (Subongkot, 2019). The injection volume for each sample was 20 µl, and the mobile phase consisted of acetonitrile:water ratio of 75:25 v/v with a flow rate of 1 ml/min. The detection wavelength of celecoxib was 250 nm.

### 2.6. Statistical analysis

All data were statistically analyzed by one-way analysis of variance (ANOVA). Values of *p* < .05 were considered statistically significant.

## 3. Results and discussion

### 3.1. Effect of sonophoresis intensity on skin penetration of celecoxib

The celecoxib concentration in receiver medium and the amounts of celecoxib which penetrated into the skin via passive penetration as a control and with the application of various sonophoresis intensities from 6 to 120 Watts/cm^2^ are shown in Table 1. The amounts of drug in the viable epidermis and dermis increased with increasing sonophoresis intensity and reached the highest amount at 30 Watts/cm^2^. However, the amounts of drug in the viable epidermis and dermis gradually decreased when the applied sonophoresis intensity was above 60 Watts/cm^2^. When the sonophoresis intensity was at 30 Watts/cm^2^, the amounts of celecoxib in the viable epidermis and dermis were significantly higher than those in the control. There were no significant differences among the celecoxib concentration in the receiver medium.

**Table 1.** Amount of celecoxib that penetrated into skin and receiver medium in the control group and in various sonophoresis intensity groups.

Formulation number	Stratum corneum (µg/cm^2^)	Viable epidermis + dermis (µg/cm^2^)	Receiver medium (µg/ml)
control	50.45 ± 28.10	18.13 ± 10.80	ND
6 W	20.38 ± 4.90*	5.24 ± 0.33	0.32 ± 0.42
8 W	26.11 ± 14.72	6.40 ± 2.60	ND
10 W	33.43 ± 20.82	10.47 ± 5.83	1.42 ± 1.47
15 W	23.27 ± 13.45*	21.15 ± 25.82	0.47 ± 0.16
30 W	34.77 ± 6.75	45.72 ± 27.50*	0.73 ± 0.25
60 W	59.87 ± 25.50	37.22 ± 15.95	1.16 ± 1.08
90 W	29.76 ± 13.39	24.07 ± 13.18	0.66 ± 0.56
120 W	27.76 ± 8.18	32.79 ± 4.13	0.45 ± 0.25

ND: not detected. Each value represents the mean ± SD (*n* = 3).

*p < .05 compared to the control.

Liu et al (Liu et al., 2006) studied the penetration of cyclosporin A in rat skin after treated with sonophoresis using different intensities at 0.4, 0.8, and 1.2 Watts/cm^2^ for 30 min. The amount of cyclosporin A in the skin increased and reached the highest amount at 0.8 Watts/cm^2^ following the increase of sonophoresis intensity. However, the amount of cyclosporin A decreased when the intensity was at 1.2 Watts/cm^2^. This result confirmed that there was a threshold level for skin penetration enhancement of sonophoresis intensity. The decrease of drug penetration when the intensity was used above the threshold level might cause the excessive generation of cavitation clouds resulting in a decrease of the energy delivered to the tissue.

According to the study by Herwadkar et al. (Herwadkar et al., 2012), the continuous use of sonophoresis for 2 min at 20 kHz with a 3 mm distance of the probe tip from the skin, an intensity of 6.9 Watts/cm^2^ and using sodium lauryl sulfate as the coupling medium could significantly increase the skin penetration of ketoprofen compared to passive penetration (∼7-fold).

Ultrasound can be classified as high-frequency ultrasound (3–10 MHz), medium-frequency ultrasound (0.7–3 MHz), and low-frequency ultrasound (18–100 kHz). Generally, low-frequency ultrasound can increase the skin penetration of drugs more than medium-frequency ultrasound (Lavon & Kost, 2004; Yin et al., 2016). Many parameters affecting the enhancement of skin penetration by sonophoresis are pretreatment time, the distance between the probe tip and skin, the type of coupling medium, the duty cycle, and sonophoresis intensity. The use of a surfactant as a coupling medium could increase the skin penetration of a drug when applying sonophoresis when compared to using water (Herwadkar et al., 2012). In this study, the distance between the probe tip and skin was 3 mm, and sonophoresis was applied continuously for 2 min (100% duty cycle) using water as the coupling medium. When the sonophoresis intensity was 30 Watts/cm^2^, the skin penetration of celecoxib was significantly increased compared to passive penetration (∼2.5-fold).

The skin penetration enhancement mechanism of sonophoresis is not yet clearly understood. It has been suggested that these skin penetration enhancement mechanisms result from thermal effects and cavitation (Park et al., 2014). Ultrasound can increase the temperature of the exposed medium, leading to enhanced permeability of the skin. However, thermal effects were not the main enhancement mechanism. Cavitation is the generation of microbubbles in a liquid medium. The stratum corneum barrier, a rate-limiting step in percutaneous absorption, occurs from the keratin inside corneocytes, intercellular lipids, and corneodesmosomes. There was evidence showing that low-frequency sonophoresis caused 1–2 micrometer-sized pores on the stratum corneum surface (Lee et al., 2010). Therefore, it is suggested that cavitation can fluidize intercellular lipids and disrupt keratin and corneodesmosomes, resulting in an increase in skin permeability.

### 3.2. Combined effects of the microemulsion and sonophoresis on the skin penetration of celecoxib

The celecoxib loaded microemulsion used in this study had an average particle size at 77.88 nm, exhibited neutral charge, and was oil-in-water microemulsion (Subongkot & Sirirak, 2020). The amount of celecoxib which penetrated into the skin and receiver medium from different treatments, which were passive penetration as a control, application of the celecoxib-loaded microemulsion (data from reference (Subongkot & Sirirak, 2020)), application of sonophoresis at an intensity of 30 Watts/cm^2^, and the combined application of sonophoresis and the celecoxib-loaded microemulsion, is shown in Table 2. The amount of celecoxib in the viable epidermis and dermis of the skin treated with only the celecoxib-loaded microemulsion was significantly higher than in the control group. However, the amount of celecoxib in the viable epidermis and dermis of the skin treated with both sonophoresis and the celecoxib-loaded microemulsion was significantly lower than in the celecoxib-loaded microemulsion group. There was no significant difference in the amount of celecoxib in the viable epidermis and dermis of the skin treated with sonophoresis alone or with the combination of sonophoresis and the celecoxib-loaded microemulsion. The celecoxib concentration in the receiver medium of skin treated with celecoxib-loaded microemulsion was significantly higher than skin treated with sonophoresis alone and skin treated both celecoxib-loaded microemulsion and sonophoresis. There was no significant difference of celecoxib concentration in the receiver medium between skin treated with sonophoresis and a combination of celecoxib-loaded microemulsion and sonophoresis. These results revealed that the combined application of the microemulsion and sonophoresis led to a decrease in the skin penetration of celecoxib. This study also found that among the microemulsion, sonophoresis, and combined microemulsion and sonophoresis techniques, the use of the microemulsion alone provided the highest skin penetration of celecoxib.

**Table 2.** Amount of celecoxib that penetrated into skin and receiver medium for each treatment.

Formulation number	Stratum corneum (µg/cm^2^)	Viable epidermis + dermis (µg/cm^2^)	Receiver medium (µg/ml)
Control	50.45 ± 28.10	18.13 ± 10.80	ND
Celecoxib loaded MC 2	97.58 ± 51.24	160.44 ± 33.32	14.26 ± 15.17*
30 W Sonophoresis	34.77 ± 6.75	45.72 ± 27.50	0.73 ± 0.25
Celecoxib loaded MC 2 + 30 W Sonophoresis	60.35 ± 27.48	65.97 ± 6.01*	0.53 ± 0.71

ND: not detected. Each value represents the mean ± SD (*n* = 3).

*p < .05 compared to the control.

### 3.3. Transfollicular penetration study

**Figure 1.**
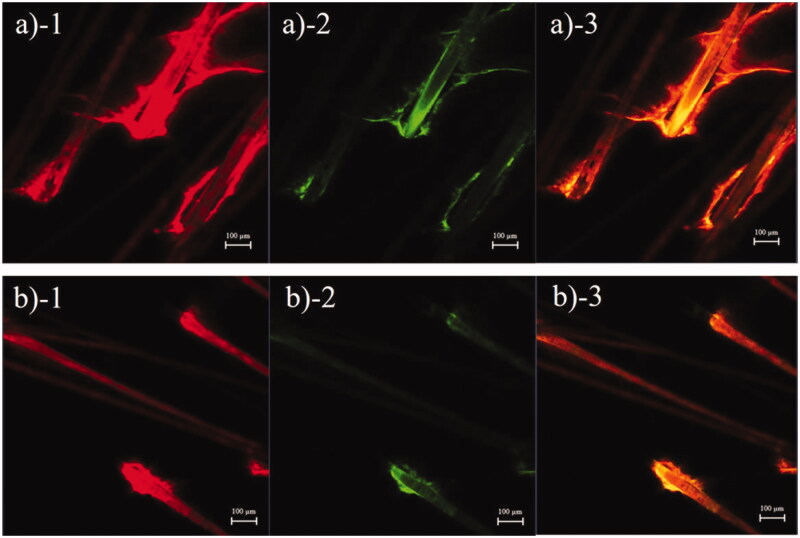
CLSM images (*x*–*y* plane) showing follicular localization of porcine skin treated with (a) a rhodamine B base-loaded, NBD-PE-labeled microemulsion and (b) sonophoresis combined with the rhodamine B base-loaded, NBD-PE-labeled microemulsion, observed at 6× magnification. Each image is divided into 3 parts: (1) red fluorescence of rhodamine B base; (2) green fluorescence of NBD-PE; (3) overlay of 1 and 2. The scale bar represents 100 µm.

The transfollicular pathway plays an essential role in skin penetration enhancement of microemulsions (Subongkot & Sirirak, 2020). To evaluate the decrease in skin penetration of celecoxib in the microemulsion when it was combined with sonophoresis, the tracking of transfollicular penetration using multifluorescent-labeled microemulsion particles was evaluated. The follicular localization of a rhodamine B base-entrapped, NBD-PE-labeled microemulsion from porcine skin treated with the microemulsion or with the combination of the microemulsion and sonophoresis as observed with low magnification are shown in Figure 1(a,b), respectively. The skin treated with only the microemulsion had stronger red and green fluorescence intensity at the hair follicles than the skin treated with combined sonophoresis and microemulsion application, which indicated that the microemulsion penetrated the skin predominantly via hair follicles. However, penetration via hair follicles of the rhodamine B base-entrapped, NBD-PE-labeled microemulsion decreased when the skin was pretreated with sonophoresis.

**Figure 2.**
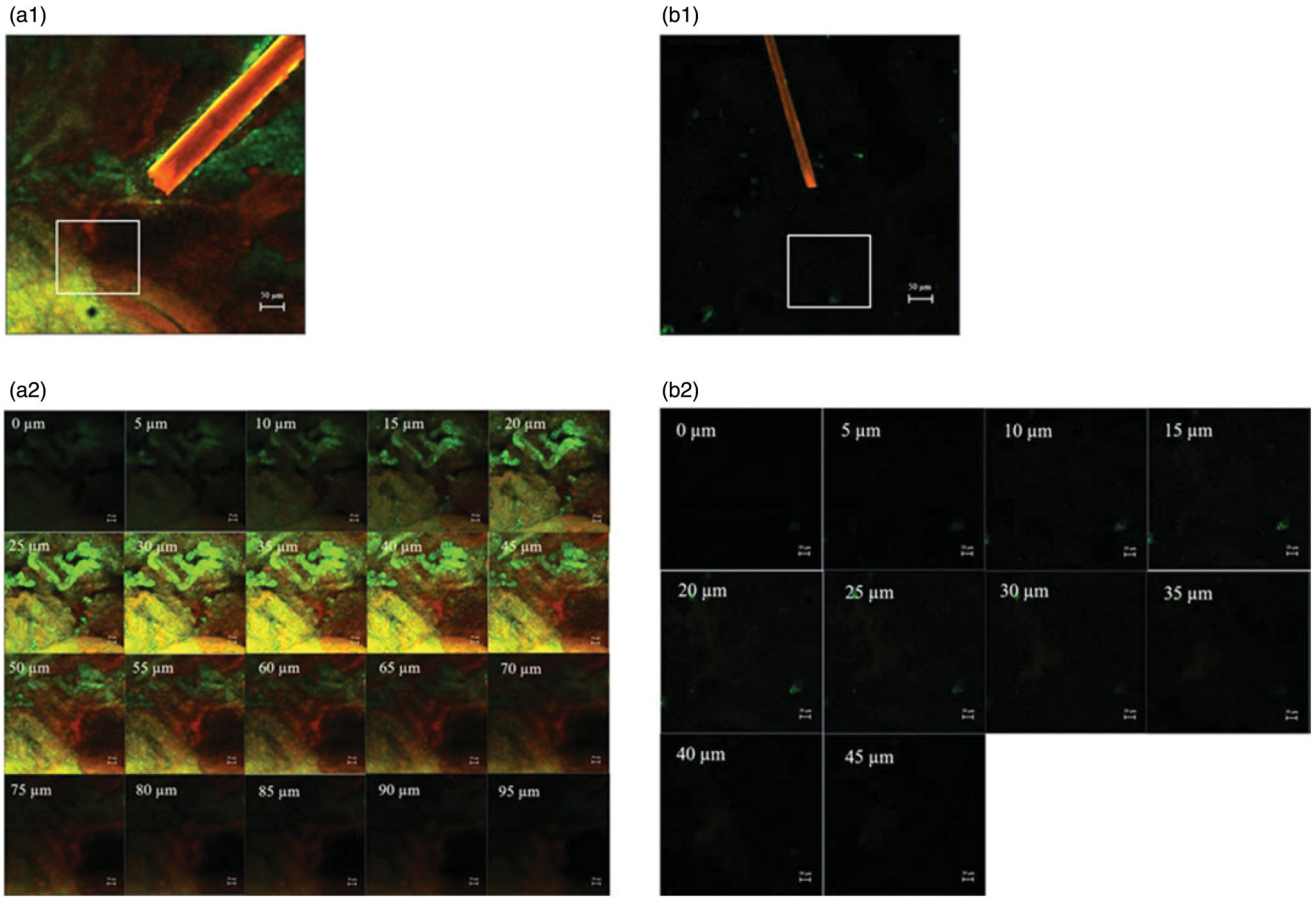
CLSM images (*x*–*y* plane) show follicular localization of porcine skin treated with (a1) a rhodamine B base-loaded, NBD-PE-labeled microemulsion and (b1) sonophoresis combined with the rhodamine B base-loaded, NBD-PE-labeled microemulsion, observed with a 10× objective lens. The scale bar represents 50 µm. (a2) and (b2) are serial *x*–*z* planes of marked areas from (a1) and (b1), respectively, at various skin depths as observed with a 20× objective lens. The scale bar represents 20 µm.

**Figure 3.**
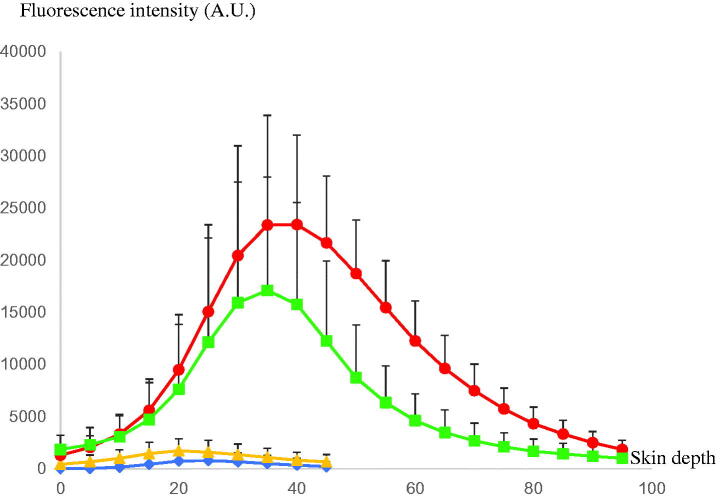
The comparison of fluorescence intensity profiles of rhodamine B base and NBD-PE at various skin depths of Figure 2(a2) (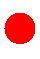 = rhodamine B base, 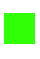 = NBD-PE) and Figure 2(b2) (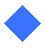 = rhodamine B base, 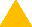 = NBD-PE). A.U.: arbitrary units. The bar represents standard deviation.

Figure 2(a1,b1) shows follicular penetration of the skin treated with only the rhodamine B base-entrapped, NBD-PE-labeled microemulsion, and that pretreated with sonophoresis followed by rhodamine B base-entrapped, NBD-PE-labeled microemulsion application, respectively, as observed with a higher magnification than in Figure 1. The serial *x*–*z* plane images of the marked areas in Figure 2(a1,b1) are shown in Figure 2(a2,b2), respectively. To quantitatively analyze the decrease in transfollicular pathway penetration of the skin pretreated with sonophoresis, the fluorescence intensities of rhodamine B base and NBD-PE in Figure 2(a2,b2) were evaluated at the middle horizontal line of each image and were plotted against different skin depths as shown in Figure 3. The fluorescence intensity profiles of rhodamine B base and NBD-PE from the skin that was pretreated with sonophoresis were significantly lower than those of the skin that was not pretreated with sonophoresis. These results indicated that the skin pretreated with sonophoresis resulted in a decrease in rhodamine B base-entrapped, NBD-PE-labeled microemulsion transfollicular penetration. This study suggested that the decrease in skin penetration of celecoxib from combined sonophoresis and microemulsion application resulted from the reduction in transfollicular pathway penetration of microemulsion particles.

The results of this study were in accordance with those reported in a study by Rangsimawong et al. (Rangsimawong et al., 2015), in which the combination of sonophoresis and liposomes application led to a decrease in the skin penetration of an entrapped drug. The authors suggested that sonophoresis might plug the hair follicle orifices, resulting in a decrease in transfollicular penetration of liposomes. Therefore, the cavitation generated from sonophoresis might have obstructed hair follicles and interfered with the penetration of microemulsion particles through the transfollicular pathway.

## 4. Conclusions

Although either sonophoresis or microemulsion application alone could increase the skin penetration of celecoxib, their combination resulted in a decrease in skin penetration. The results of the CLSM study using the colocalization technique of multifluorescently labeled particles suggested that the reduction in skin penetration of celecoxib from combined sonophoresis and microemulsion application was caused by a decrease in transfollicular penetration, which is the major skin absorption pathway of microemulsions.
